# Melanin Transfer in Human 3D Skin Equivalents Generated Exclusively from Induced Pluripotent Stem Cells

**DOI:** 10.1371/journal.pone.0136713

**Published:** 2015-08-26

**Authors:** Karl Gledhill, Zongyou Guo, Noriko Umegaki-Arao, Claire A. Higgins, Munenari Itoh, Angela M. Christiano

**Affiliations:** 1 Department of Dermatology, Columbia University, New York, NY, United States of America; 2 Department of Genetics and Development, Columbia University, New York, NY, United States of America; University Hospital Hamburg-Eppendorf, GERMANY

## Abstract

The current utility of 3D skin equivalents is limited by the fact that existing models fail to recapitulate the cellular complexity of human skin. They often contain few cell types and no appendages, in part because many cells found in the skin are difficult to isolate from intact tissue and cannot be expanded in culture. Induced pluripotent stem cells (iPSCs) present an avenue by which we can overcome this issue due to their ability to be differentiated into multiple cell types in the body and their unlimited growth potential. We previously reported generation of the first human 3D skin equivalents from iPSC-derived fibroblasts and iPSC-derived keratinocytes, demonstrating that iPSCs can provide a foundation for modeling a complex human organ such as skin. Here, we have increased the complexity of this model by including additional iPSC-derived melanocytes. Epidermal melanocytes, which are largely responsible for skin pigmentation, represent the second most numerous cell type found in normal human epidermis and as such represent a logical next addition. We report efficient melanin production from iPSC-derived melanocytes and transfer within an entirely iPSC-derived epidermal-melanin unit and generation of the first functional human 3D skin equivalents made from iPSC-derived fibroblasts, keratinocytes and melanocytes.

## Introduction

Human 3D skin equivalents (HSEs) are *in vitro* models used to conduct experiments on processes involving the skin e.g. disease progression and drug discovery. They are widely used for several reasons, but mainly because many studies report differences in phenotype, cellular signaling, cell migration, and drug responses when the same cells are grown under 2D vs 3D conditions [1–4]. When lifted to the air interface HSEs differentiate and stratify, recapitulating the complex and dynamic environment found in human skin, meaning that data generated using these models is often fairly representative of an *in vivo* response. Currently however, the utility of HSEs is limited since these models lack the full cellular complexity of human skin i.e. they contain few cell types and no appendages [5].

One major reason for this is that these models consist of cells isolated from freshly discarded tissue following surgery. This tissue is often small and therefore it is usually only possible to isolate and expand in culture the most numerous cell types i.e. dermal fibroblasts and epidermal keratinocytes. Although many other skin cell types can be isolated from such tissue it is likely at the expense of these aforementioned critical components. Moreover, many minority skin cell subpopulations cannot be greatly expanded in culture e.g. Merkel cells [6]. One possible solution to this problem is to generate HSEs from skin cells isolated from tissue harvested from multiple individuals. However, in certain contexts such as drug discovery, subtle inter-individual effects are often noted [7], and therefore skin constructs established from cells from multiple individuals can greatly complicate data analysis. Additionally, many cell types found in skin, such as macrophages, are only transient and enter the skin in response to a pathological condition [8]. In order to isolate large numbers of these cells to include these cells in HSEs and accurately model cutaneous disorders, biopsies are required from patients who often are unwilling to undergo any procedure that may exacerbate their condition.

The discovery of induced pluripotent stem cells (iPSCs) in 2006 [9] was a major breakthrough for biomedical science. Moreover, the recent ability to generate iPSCs using integration-free approaches has resulted in their increased use in translational research since these cells and their progeny do not contain viral DNA [10,11]. iPSCs offer an approach to circumvent many of the issues associated with current HSEs because of their unlimited growth and differentiation potential. Additionally, since cells differentiated from an iPSC are expected to retain characteristics of the original donor, such as disease phenotype [12], and since iPSCs can be created from less invasive samples such as blood [13], iPSCs offer an alternative source of starting cells for modeling skin disorders.

Many hurdles still exist before iPSC-derived HSEs can reach a level of complexity nearing that of human skin. Many of the protocols required to differentiate an iPSC into other skin cell types, such as Langerhans cells and Merkel cells, have yet to be developed. Additionally, cost, retention of epigenetic memory, genomic instability and lack of complex structures like appendages are other factors which remain to be fully addressed.

Our group first demonstrated the feasibility of iPSC-derived HSEs for the study of normal skin homeostasis and disease. We created the simplest version of a normal HSE consisting solely of iPSC-derived fibroblasts and keratinocytes [14]. Additionally, we showed that the skin blistering disease recessive dystrophic epidermolysis bullosa could be modeled in HSEs comprised of iPSC-derived keratinocytes and normal fibroblasts [12]. In this study, our goal was to increase the complexity of these HSEs by adding additional iPSC-derived cell types, to better recapitulate normal human skin. Epidermal melanocytes represent the second most abundant cell type found in normal human epidermis and are largely responsible for both skin color and protection against the damaging effect of ultraviolet radiation via production of melanin [15]. Moreover, pigmentation changes are often observed in response to disease, or drug treatments, making HSEs that incorporate melanocytes an important tool in the drug discovery process. Since differentiation protocols already exist for generating melanocytes from stem cells [16–20] they represent a logical addition to our iPSC-derived HSEs.

In this report, we have generated the first HSEs from iPSC-derived keratinocytes, melanocytes and fibroblasts containing a functional iPSC-derived epidermal-melanin unit. Further, we report the ability of iPSC-derived keratinocytes to functionally participate in melanin uptake and transfer. With these findings, we have generated functional HSEs derived solely from iPSCs that more fully recapitulate human *in vivo* skin.

## Materials and Methods

### Cell Culture

Epidermal melanocytes, keratinocytes and fibroblasts were isolated from neonatal foreskin from healthy individuals from the Children’s Hospital at Columbia University Medical Center. Samples were de-identified prior to being received by researchers and designated as nonhuman subject research under 45 CFR Part 46, and we therefore received an Institutional Review Board exemption at Columbia University to use these materials. Epidermal melanocytes were cultured (after selective trypsinization of keratinocytes and after removal of contaminating fibroblasts with 150μg/ml geneticin sulphate (G418) (Life Technologies) in 48 hour cycles at the p0 to p1 stage of primary culture [21] in Clonetics MGM-4 Melanocyte Growth Media-4 with 100nM endothelin-3 (EDN3) (Lonza). Any residual and contaminating keratinocytes were removed by a further round of selective trypsinization. iPSC-derived melanocytes were cultured in fibronectin-coated culture flasks (Discovery Labware) with iPSC-melanocyte media (S1 Fig). Normal epidermal keratinocytes and iPSC-derived keratinocytes were cultured in CnT-07 media (CELLnTEC). Normal dermal fibroblasts, iPSC-derived fibroblasts and Melanoma cells (MNT-1) (a kind gift from Dr. Vincent Hearing) were cultured in fibroblast medium (DMEM culture media (Life Technologies) supplemented with 10% fetal bovine serum (FBS) (Life Technologies) and 1% penicillin-streptomycin (Life Technologies)). Cells were incubated at 37°C in a 5% CO_2_ atmosphere and media was replenished every second or third day.

### iPSC Generation and Characterization

We generated and characterized iPSC lines as previously described [12,14]. Briefly, human fibroblasts were isolated from normal foreskin and expanded in fibroblast medium. These cells were transduced by pMXs-based retroviruses with four transcription factors: c-MYC, SOX2, OCT4, and KLF4. After 6 days, the transduced cells were reseeded on mitomycin C-treated SNL feeder layers in human embryonic stem cell medium (Knockout (KO)-DMEM supplemented with 20% KO-Serum Replacement (Life Technologies), 1% GlutaMax-I (Life Technologies), 1% nonessential amino acid (Life Technologies), 1% penicillin-streptomycin, 0.18% β-mercaptoethanol (Life Technologies) and 4ng/ml basic fibroblast growth factor (FGF_2_) (R&D Systems)) until colonies appeared. Colonies of iPSCs were mechanically picked up and replaced on mitomycin C-treated SNL feeder layers for expansion and characterization.

### Fibroblast Differentiation from iPSCs

iPSC-derived fibroblasts used in this study were generated by a modification of our protocol as previously described [14]. Briefly, we generated embryoid bodies in KO serum replacement media without FGF_2_ and supplemented with 0.3mM ascorbic acid (Sigma), 10ng/ml transforming growth factor β-2 (TGFβ2) (R&D Systems) and ITS-A supplement (Life Technologies) on a low-binding dish. For inducing cell outgrowth, embryoid bodies were attached to a gelatin-coated dish, and cultured in DMEM (with high glucose) (Life Technologies) supplemented with ascorbic acid and 20% FBS for 10 days. Cells that grew out from embryoid bodies were fed every other day and passaged weekly for maintenance and expansion.

### Keratinocyte Differentiation from iPSCs

iPSC-derived keratinocytes used in this study were generated by a modification of our protocol as previously described [12]. Briefly, small clumps of iPSCs were subcultured on Matrigel (Fisher Scientific) in SNL-conditioned knockout serum replacement media for 1 day. iPSCs were incubated in defined keratinocyte serum-free media (Life Technologies) supplemented with 1μM all-trans retinoic acid (Sigma) and 10ng/ml bone morphogenetic protein-4 (BMP4) (R&D Systems) for 4 days. Media was changed to CnT-07, and differentiated iPSCs were maintained in culture for 30 days. Cells were fed every other day and passaged weekly for maintenance and expansion.

### Melanocyte Differentiation from iPSCs

Melanocytes were differentiated from iPSCs using a combination of established protocols [18,20] (S1 Fig). Briefly, we generated embryoid bodies in KO-DMEM supplemented with 20% KO-Serum Replacement, 1% GlutaMax-I, 1% nonessential amino acid, 1% penicillin-streptomycin, 500nM LDN193189 (Stemgent) and 10μM SB431542 (Tocris Bioscience) for 3 days followed by 50% KO-DMEM supplemented with 20% KO-Serum Replacement, 1% GlutaMax-I, 1% nonessential amino acid and 1% penicillin-streptomycin and 50% Neurobasal Media (Life Technologies) with 2% B-27 Supplement (Life Technologies), 2% N-2 supplement (Life Technologies), 1% GlutaMax-I, 100nM EDN3, 25ng/ml BMP4 and 50ng/ml stem cell factor (SCF) (R&D Systems) for 3 days. On day 6 embryoid bodies were attached to feeder-free fibronectin-coated culture flasks in Neurobasal Media with 2% B-27 Supplement, 2% N-2 supplement, 1% GlutaMax-I, 100nM EDN3, 25ng/ml BMP4 and 50 ng/ml SCF and cells that grew out were fed every other day and passaged weekly for maintenance and expansion until differentiation was achieved (day 28). From day 2 and 14 onwards, 3μM CHIR99021 (Stemgent) and 500μM N(6),2'-O-dibutyryladenosine 3':5' cyclic monophosphate (dbcAMP) (Sigma), respectively, were added to the media.

### Immunofluorescence

Cells were pre-fixed on gelatin-coated chamber slides (Fisher Scientific) by adding an equal volume of 4% paraformaldehyde (PFA) (Sigma) to culture media for 2 minutes at room temperature (RT). Pre-fix solution was removed and cells were fixed with 4% PFA for 10 minutes, washed briefly with phosphate buffered saline (PBS) (Life Technologies) and permeabilized with 0.1% Triton-X-100 (Fisher Scientific) for 10 minutes. Formalin fixed, paraffin wax embedded tissue was cut (7μm) onto poly-L-lysine-coated slides (Fisher Scientific), dried overnight at 55°C, dewaxed in xylene and rehydrated through a graduated ethanol series (100%, 95%, 70%) and distilled water (dH_2_O). Antigen retrieval was performed by boiling slides in 10mM sodium citrate buffer (pH 6.0) for 15 minutes in a microwave oven. The slides were left to cool to RT for 20 minutes before removal. Samples were rinsed briefly with PBS and non-specific binding was blocked using 1.5% fish skin gelatin (Sigma) in PBS containing 0.025% Triton-X-100 for 90 minutes at RT. Samples were incubated with primary antibodies (MITF-M, 1:500, Abcam, SOX-10, 1:100, Santa Cruz Biotechnology, Keratin 1, 1:1000, Covance, gp-100, 1:100, Monosan, Keratin-14, 1:1000, Covance, Loricrin, 1:500, Covance) overnight at 4°C. After appropriate washing with PBS, samples were incubated with fluorophore-conjugated secondary antibodies (Donkey anti-Rabbit 488, 1:500, Invitrogen, Goat anti-Mouse 594, 1:500, Invitrogen, Donkey anti-Goat 594, 1:500, Invitrogen, Donkey anti-Mouse 488, 1:500, Invitrogen) for 1 hour at RT. Slides were coverslipped using Vectashield mounting media containing 4',6-diamidino-2-phenylindole (DAPI) (Vectashield) and samples were examined using a Zeiss LSM 5 Exciter confocal laser scanning microscope.

### qRT-PCR

RNA was extracted using an RNeasy Mini Kit (Qiagen), and DNA was removed by DNase treatment (Invitrogen) to avoid genomic DNA amplification. cDNA was synthesized using 1μg RNA by SuperScript III reverse transcriptase and Oligo-dT primer (Invitrogen) according to the manufacturer's instructions. PCR reactions were performed with Platinum PCR SuperMix (Invitrogen). Quantitative PCR was performed on an ABI 7300 machine and analyzed with ABI Relative Quantification Study software (Applied Biosystems). Primers were designed according to ABI guidelines, and all reactions were performed using Power SYBR Green PCR Master Mix (Applied Biosystems). The following protocol was used: step 1, 50°C for 2 minutes; step 2, 95°C for 10 minutes; step 3, 95°C for 15 seconds; step 4, 60°C for 1 minute; repeat steps 3 and 4 for 40 cycles. All samples were run in triplicate for three independent runs and normalized against an endogenous internal control, GAPDH. All primer sequences are supplied in S1 Table.

### Fluorescent Bead Uptake Experiments

Normal or iPSC-derived keratinocytes were seeded onto gelatin-coated four-well chamber slides at a cell density of 2 × 10^4^ cells/well in CnT-07 media. Cultures were allowed to expand until they were about 70% confluent. Cells were incubated with freshly sonicated fluorescent microspheres 0.5μm (red) in diameter (Life Technologies) (previously demonstrated to be an appropriate model for melanosome uptake in epidermal keratinocytes [22]). Microspheres were pre-incubated for 1 hour at 37°C in CnT-07 media (containing 10% FBS) before being incubated with cells for different time points (0, 2, 4 or 6 hour) at a final concentration of 288,000 particles/ml in CnT-07 media. Media was removed and cells were washed 3 times in fresh cold media followed by a final wash in cold PBS to remove non-ingested microspheres. Cells were then fixed in ice-cold methanol (Fisher Scientific) for 10 minutes at RT, rinsed with PBS, coverslipped using Vectashield mounting media containing DAPI and examined using a Zeiss LSM 5 Exciter confocal laser scanning microscope. Quantitative analysis of the beads was performed by counting the number of internalized beads in 30 cells for each time point, randomly taken from 3 microscopic fields (10 cells per microscopic field) in 3 different experiments (resulting in 90 cells analyzed per condition), and values are expressed as the mean value ± SEM. Statistical analysis was performed using the Student’s *t* test and significance level has been identified as p<0.05. In order to determine that only internalized beads were counted in the quantitative analysis we performed parallel phase contrast and fluorescence microscopy.

### Melanosome Isolation and Uptake Experiments

The isolation of melanosomes for transfer was performed as previously described [23] with a few modifications. Briefly, MNT-1 human melanoma cells were cultured in T75 tissue culture flasks (Fisher Scientific). After reaching 95% confluence (approximately 14.5 x 10^6^ cells per flask), the cells were harvested with trypsin-EDTA (Life Technologies), washed with PBS, and stored in a freezer until used. After thawing the frozen MNT-1 cells, 1ml of cold lysis buffer (0.1M Tris-HCl (Invitrogen) pH 7.5, 1% Igepal CA-630 (Sigma), 0.01% SDS (Sigma)) was added to the cells obtained from each T75 tissue culture flask and homogenized on ice using 20 strokes in 2 minutes with a Dounce glass/glass homogenizer. The homogenate was then stored at 4°C for 20 minutes with mixing every 10 minutes. After centrifugation (1 × 10^3^g for 10 minutes at 4°C), the supernatants were transferred to new Eppendorf tubes (1.5ml) and centrifuged again in the same manner. The supernatants were further centrifuged (2 × 10^4^g for 10 minutes at 4°C) and the precipitates were washed twice by PBS with brief and gentle mixing in order to avoid dispersion and were centrifuged again (2 × 10^4^g for 10 minutes at 4°C). The pellets were then used as the melanosome-rich fraction.

To prepare a dispersion of melanosomes, 100μl of CnT-07 media was added to each melanosome pellet (obtained from a T75 tissue culture flask) and was mixed by pipetting. When the melanosomal suspension was homogeneous, 50μl was added to each normal or iPSC-derived keratinocyte containing gelatin-coated well of a 6-well plate (Fisher Scientific) for 24 hours. For experiments involving soybean trypsin inhibitor (STI) (Sigma), an inhibitor of keratinocyte phagocytosis [24], 1mg/ml STI was added to the cells 2 hours before addition of the melanosomes and kept in the media for the duration of the 24 hour experiment. Keratinocytes adherent to the plate were washed 2 times with PBS to remove non-ingested melanosomes and were then processed for Fontana-Masson staining.

### Fontana-Masson Staining

Normal or iPSC-derived melanocytes were washed twice with PBS and then fixed with ice-cold methanol for 10 minutes at RT. After washing the cells twice with dH_2_O, they were incubated with Fontana ammoniacal silver solution (American MasterTech) for 1 hour at 37°C. After further washing the cells twice with dH_2_O, they were examined by light microscopy using a Zeiss Axiovert 25 microscope. For staining of 3D skin equivalents the Fontana-Masson staining kit (American MasterTech) was used exactly as described in the manufacturer’s instructions. Quantification of Fontana-Masson staining was calculated using the analyze particles function of ImageJ. We counted 27 cells for each condition, randomly taken from 3 microscopic fields (9 per microscopic field) in 3 different experiments (resulting in 81 cells analyzed per condition), and values are expressed as the mean value ± SEM. Statistical analysis was performed using the Student’s *t* test and significance level has been identified as p<0.05.

### Co-Culture Experiments

For co-culture studies using normal or iPSC-derived melanocytes with normal or iPSC-derived keratinocytes; keratinocytes were first seeded onto gelatin-coated eight-well chamber slides at a cell density of 3.6 x 10^4^ cells/well in CnT-07 media. 24 hours later 4 x 10^3^ melanocytes were then added to each well in CnT-07 media. The co-cultures were left for a further 7 or 24 hours before being examined by immunofluorescence.

### Generation of 3D Skin Equivalents

For melanocyte localization experiments, 3D skin equivalents were generated by adapting a previously described protocol [25]. Briefly, a type I collagen matrix (containing 0.5 x 10^6^ iPSC-derived fibroblasts) was deposited onto polyethylene terephthalate membranes (BD Biosciences), and allowed to polymerize. After incubation of the polymerized matrix for 7 days, 1 x 10^6^ iPSC-derived keratinocytes and 0.1 x 10^6^ iPSC-derived melanocytes were seeded onto the matrix, and incubated for a further 7 days. The composite culture was raised to the air-liquid interface and fed from below to induce epidermal differentiation. 3D skin equivalents were harvested 14 days later and either snap frozen in LN_2_ or embedded in wax. For melanin quantification studies involving 40 μM forskolin where we were aiming to determine the functionality of our iPSC-derived melanocytes in a 3D environment, the protocol was modified such that forskolin was added to the cell culture media at the same time as the epidermal cells were added to the skin construct (21 days in total) and that the ratio of iPSC-derived keratinocytes to iPSC-derived melanocytes was 1:1. Although this ratio was unlikely to result in a physiological number of melanocytes at the basal layer of the epidermis, these conditions have previously been shown to produce optimal results when studying the effects of drugs on melanogenesis in conventional 3D skin equivalents [26].

### Hematoxylin and Eosin Staining

Following dewaxing, 7μm sections were stained with Mayer’s Hematoxylin (Sigma) at RT for 3 minutes. Blueing was achieved by rinsing in tap water while differentiation was achieved by rinsing in 1% acid ethanol. Counterstaining was achieved by rinsing with eosin (Sigma) for 30 seconds while dehydration was achieved by sequential washing with 95% ethanol, 100% ethanol and Histo-Clear (National Diagnostics). Slides were coverslipped with DPX (Agar Scientific) and examined by light microscopy using a Zeiss Axioplan 2 microscope.

### Melanin Assay

Quantification of melanin in iPSC-derived 3D skin equivalents was performed as described previously [27]. Briefly, frozen 3D skin constructs were thawed at room temperature and melanin was extracted using Solvable (PerkinElmer). A 1 mg/ml melanin standard stock solution was prepared by dissolving synthetic melanin (Sigma) in Solvable and a series of melanin standards were prepared from the stock. Solvable was used as the 0.0 mg/ml standard. The tissue samples and the melanin standard series were incubated for at least 16 hours at 60±2°C in a dry bath. The extracted tissue samples and the melanin standards were cooled and centrifuged. Samples or standards were then transferred to the appropriate wells of a 96-well plate (BD Biosciences), and Solvable was added to the wells designated as blanks. The absorbance at 490 nm of each well was measured with a Molecular Devices Vmax plate reader (Molecular Devices). The effect of forskolin on pigmentation in the 3D skin equivalents was determined in triplicate in 3 different experiments. Values are expressed as the mean % increase in melanin above control ± SEM. Statistical analysis was performed using the Student’s *t* test and significance level has been identified as p<0.05.

## Results

### Generation and Characterization of iPSC-derived melanocytes

**Fig 1:**
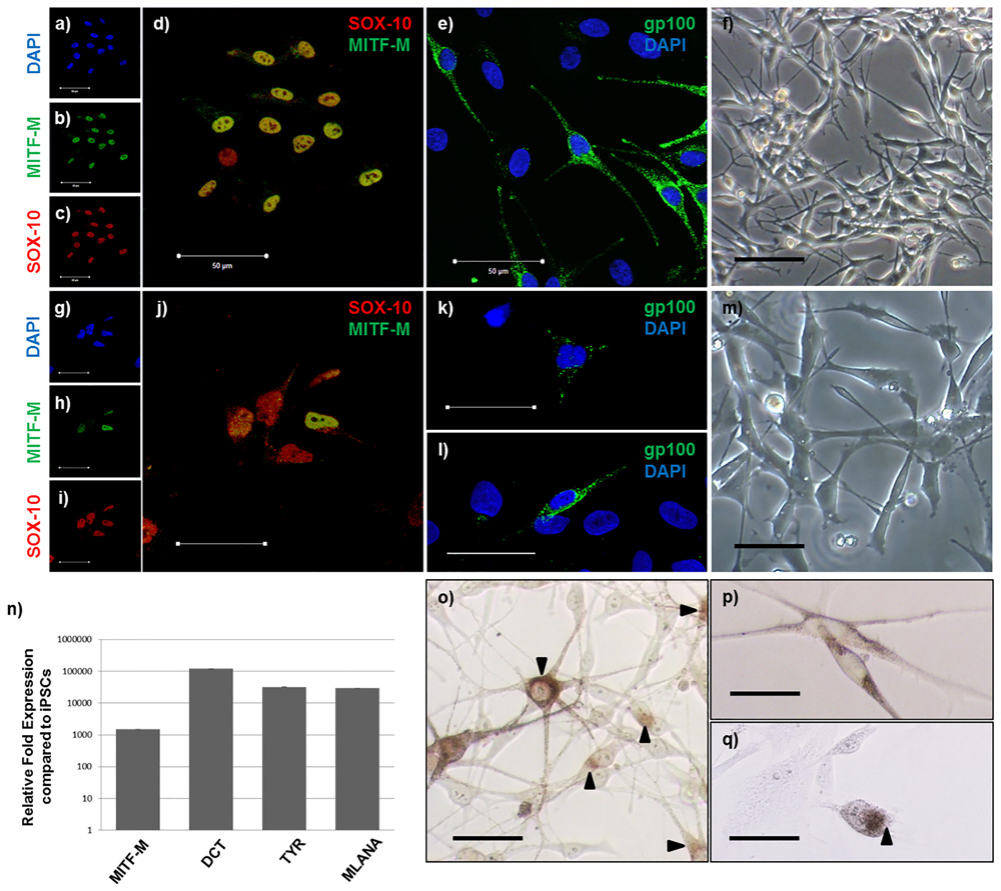
iPSC-derived melanocytes express normal melanocyte markers and produce melanin. **(a-e)** Normal epidermal melanocytes (NEM) and **(g-k)** iPSC-derived cells (at day 11 of the differentiation protocol) express SOX-10, MITF-M and gp-100. Phase contrast images of normal **(f)** and iPSC-derived **(m)** cells (at day 11 of the differentiation protocol) showing typical melanocyte morphology. Image **(l)** represents gp-100 expression in iPSC-derived cells at day 25 of the differentiation protocol. **(n)** At day 17 of the differentiation protocol expression of melanocyte specific markers is greatly up-regulated in iPSC-derived cells compared to iPSCs. **(o)** NEM produce melanin as shown by Fontana-Masson staining. **(p)** & **(q)** Brightfield microscopy and Fontana-Masson staining, respectively, of iPSC-derived cells (at day 28 of the differentiation protocol) containing melanin. Scale bar = 50μm. Arrows point to melanin as determined by Fontana-Masson staining.

We have previously reported methods and protocols to generate 3D skin equivalents from iPSC-derived fibroblasts and keratinocytes [14]. In order to generate iPSC-derived melanocytes, we adapted existing protocols [18,20] (S1 Fig). Normal epidermal melanocytes express SOX-10 (Fig 1c and 1d), MITF-M (Fig 1b and 1d) and gp-100 (Fig 1e), exhibit a classical dendritic morphology (Fig 1f) and produce melanin (Fig 1o). At day 11 of the differentiation protocol, iPSC-derived cells were positive for SOX-10 expression (Fig 1i and 1j) suggesting successful neural crest induction. Moreover, some of these SOX-10 positive iPSC-derived cells were also positive for MITF-M expression (Fig 1h and 1j) suggesting that they were melanocyte precursors (melanoblasts). Even at this early stage of the differentiation protocol, a small proportion of iPSC-derived cells were positive for gp-100 expression (Fig 1k) and had begun to adapt a typical melanocyte morphology (Fig 1m) suggesting that some melanoblasts had begun to mature into normal melanocytes. However, it was not until day 25 of the differentiation protocol that the level of gp-100 expression (as measured by immunofluorescence) in the iPSC-derived cells (Fig 1l) was similar to that of normal melanocytes (Fig 1e). We confirmed the expression of MITF-M in the iPSC-derived cells at day 17 of the differentiation protocol by qRT-PCR and determined that these cells expressed additional normal melanocyte markers such as DCT, TYR and MLANA (Fig 1n). By day 28 of the differentiation protocol, it was confirmed that the iPSC-derived cells contained melanin (Fig 1p and 1q).

### Internalization of melanosome-sized particles by iPSC-derived keratinocytes

**Fig 2:**
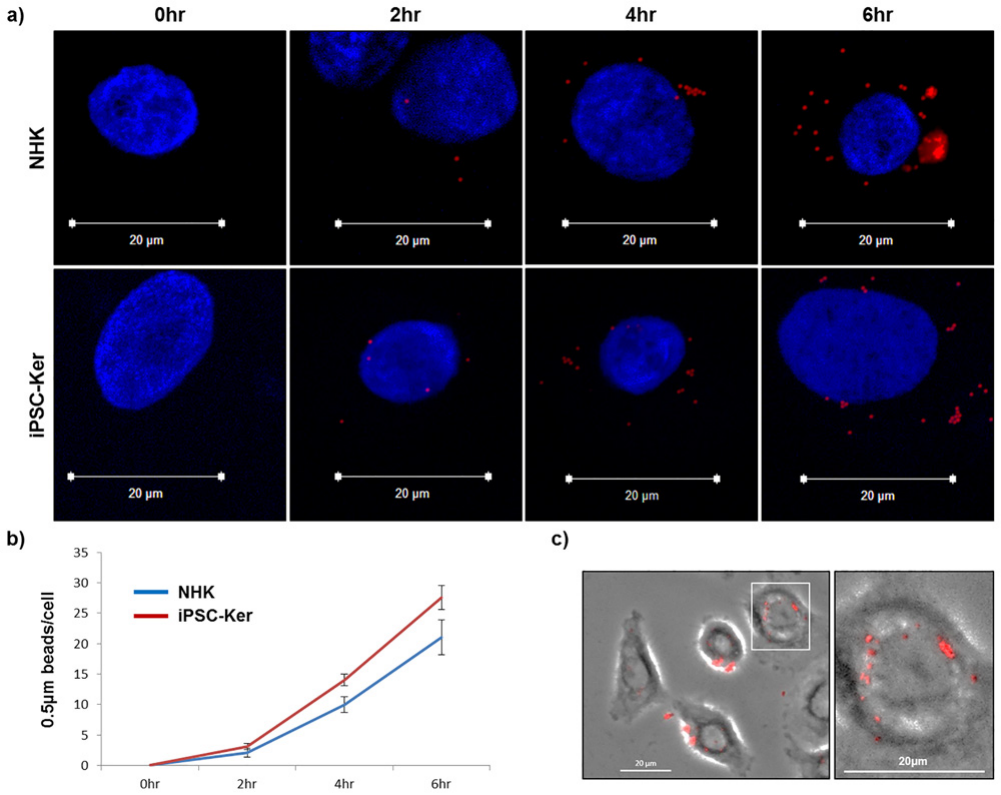
iPSC-derived keratinocytes can internalize and intracellularly transport microspheres 0.5μm in diameter. **(a)** Normal epidermal keratinocytes (NHK) and iPSC-derived keratinocytes (iPSC-Ker) co-cultured with red fluorescent microspheres 0.5μm in diameter for different time points (0, 2, 4 or 6 hours). **(b)** Quantitative analysis of microsphere internalization. **(c)** Parallel fluorescence and phase contrast microscopy showing method used to determine that only internalized beads were counted in the quantitative analysis. Blue (DAPI), Red (internalized 0.5μm microspheres).

In previous reports, we showed that our iPSC-derived keratinocytes can generate a stratified epidermis [12,14]. However, in order to generate a functional iPSC-derived epidermal-melanin unit, we investigated whether these cells could participate in the melanin transfer process. Normal melanosomes range in diameter from 0.1–1μm and the median melanosome diameter is 0.5μm [28]. Therefore, iPSC-derived keratinocytes were co-cultured with red fluorescent microspheres (0.5μm in diameter) to investigate the capability of these cells to internalize melanosome-sized particles and to determine how the efficiency of this process compared to that in normal epidermal keratinocytes. At all time points investigated, the uptake of the microspheres in normal and iPSC-derived keratinocytes was not significantly different from one another (Fig 2a and 2b). Moreover, as the length of incubation with the microspheres increased from 2 to 6 hours, the microspheres began to self-organize into a typical supranuclear cap configuration, representing a characteristic internal localization pattern that allows melanin to protect DNA within the nucleus from the damaging effects of ultraviolet radiation (Fig 2a and 2c) [29,28,30].

### Internalization of freshly isolated melanosomes by iPSC-derived keratinocytes

**Fig 3:**
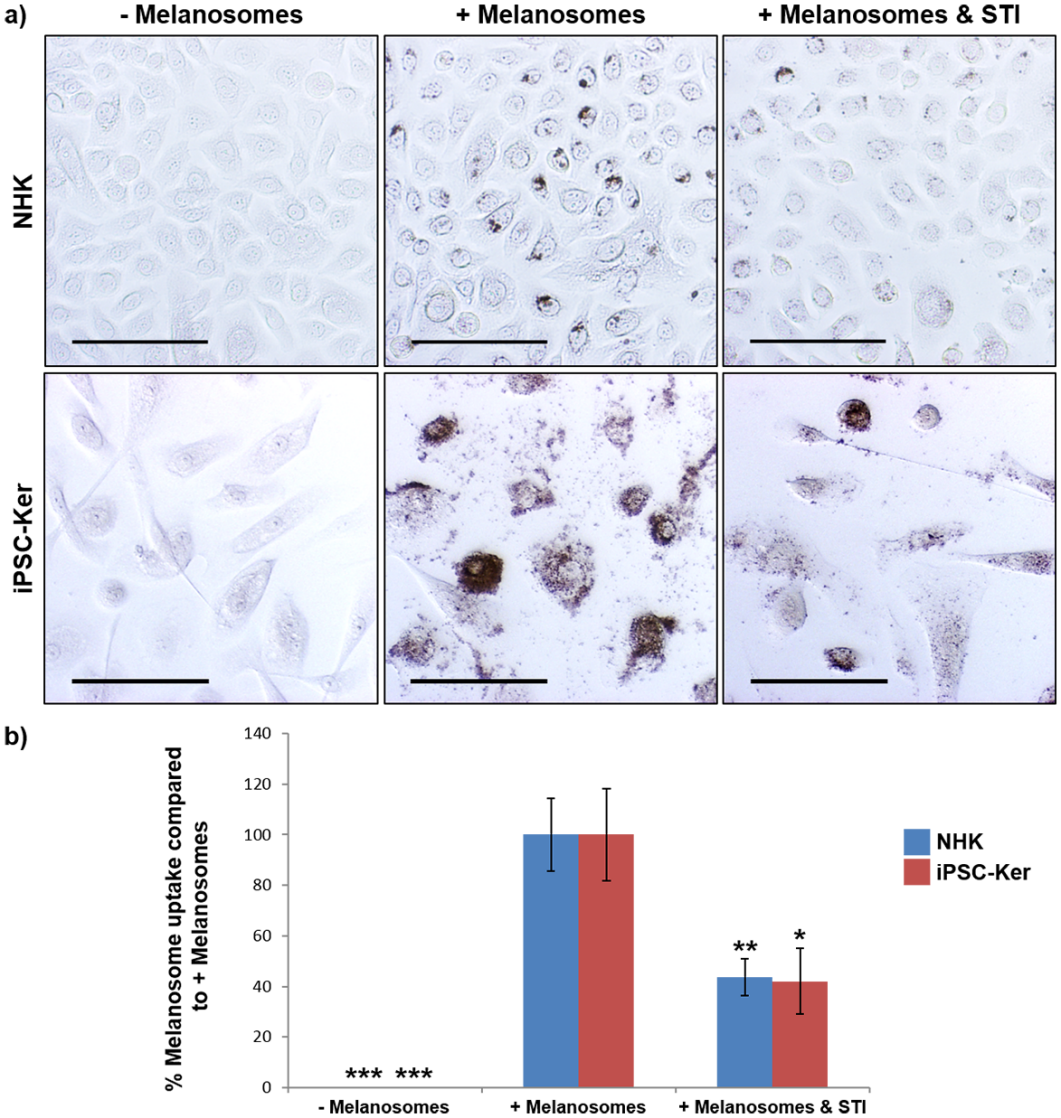
iPSC-derived keratinocytes internalize melanosomes in a PAR-2-dependent manner. **a)** Normal epidermal keratinocytes (NHK) and iPSC-derived keratinocytes (iPSC-Ker) internalize freshly isolated melanosomes within 24 hours via a mechanism involving PAR-2.–Melanosomes (Fontana-Masson stain of normal and iPSC-derived keratinocytes after 24 hours in culture without the addition of melanosomes), + Melanosomes (Fontana-Masson stain of normal and iPSC-derived keratinocytes after 24 hours in culture with the addition of melanosomes), + Melanosomes & STI (Fontana-Masson stain of normal and iPSC-derived keratinocytes after 24 hours in culture with the addition of melanosomes and soybean trypsin inhibitor). Scale bar = 100μm. **b)** Quantification of Fontana-Masson staining in a). * = p<0.05, ** = p<0.01, *** = p<0.001.

To further investigate the capability of our iPSC-derived keratinocytes to participate in the melanin transfer process, we incubated these cells with freshly isolated melanosomes (Fig 3a). Following a 24 hour incubation with these organelles, we confirmed by Fontana-Masson staining that they had been internalized by the iPSC-derived keratinocytes. Moreover, it was also clear that the melanosomes had been correctly transported within the cells, resulting in their hallmark supranuclear localization. Protease-activated receptor-2 (PAR-2) is known to be involved in melanosome phagocytosis in normal epidermal keratinocytes [31]. Therefore, in order to provide mechanistic insight into how the melanosomes had been internalized by the iPSC-derived keratinocytes, we repeated the 24 hour melanosome incubation in the presence of soybean trypsin inhibitor (STI), a known PAR-2 inhibitor [24]. STI reduced melanosome uptake by around 50% in both the normal and iPSC-derived keratinocytes (Fig 3a and 3b) confirming the involvement of PAR-2 in this process. Surprisingly, iPSC-derived keratinocytes appeared to internalize melanosomes more efficiently than normal keratinocytes, as determined by more extensive Fontana-Masson staining (Fig 3a).

### Melanin transfer between iPSC-derived melanocytes and iPSC-derived keratinocytes

**Fig 4:**
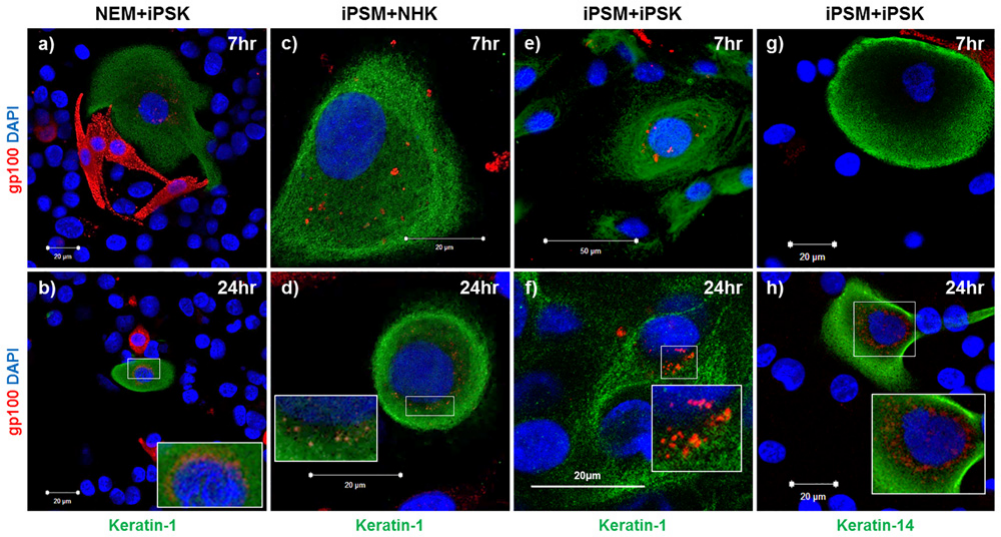
iPSC-derived melanocytes and keratinocytes participate in melanin transfer. **(a-b)** normal epidermal melanocytes (NEM) and iPSC-derived keratinocytes (iPSK) in co-culture for 7 and 24 hours. **(c-d)** iPSC-derived melanocytes (iPSM) and normal epidermal keratinocytes (NHK) in co-culture for 7 and 24 hours. **(e-h)** iPSC-derived melanocytes and iPSC-derived keratinocytes in co-culture for 7 and 24 hours. Inset (enlarged images showing transferred melanin in the perinuclear region).

We next investigated the capability of our iPSC-derived cell types to synthesize and transfer melanin, characteristic of the epidermal-melanin unit in normal human skin. Normal epidermal melanocytes were co-cultured with iPSC-derived keratinocytes (Fig 4a and 4b) and gp-100 expression was investigated to determine the ability of the latter cell type to take up melanin in a 2D context. gp-100 has previously been shown to be a useful marker in studying melanosome transfer in melanocyte/keratinocyte co-cultures [32]. After 7 hours in co-culture, the keratin-1 (suprabasal epidermal marker) positive iPSC-derived keratinocytes were also positive for gp-100 staining. At this time point, gp-100 staining was somewhat diffuse and localized throughout the cytoplasm (Fig 4a). However, by 24 hours in co-culture gp-100 staining in iPSC-derived keratinocytes was concentrated in the perinuclear region of the cell (Fig 4b), suggesting that the melanosomes had taken up a supranuclear cap arrangement.

Next, iPSC-derived melanocytes were co-cultured with normal epidermal keratinocytes (Fig 4c and 4d) to investigate their ability to synthesize and transfer melanin in this context. After 7 hours in co-culture the keratin-1 positive normal epidermal keratinocytes were also positive for gp-100 staining suggesting that the iPSC-derived melanocytes had successfully made and transferred melanin (Fig 4c). Again, after 24 hours in co-culture the melanosomes had taken up the supranuclear cap arrangement in the normal epidermal keratinocytes (Fig 4d). Finally, iPSC-derived melanocytes and iPSC-derived keratinocytes were co-cultured (Fig 4e–4h) to investigate whether the desired abilities these cell types had displayed with their normal counterparts would be retained. After 7 and 24 hours in co-culture results closely resembled those in Fig 4a–4d suggesting that our iPSC-derived cells would be capable of making a functional epidermal-melanin unit when placed in a 3D skin equivalent. Moreover, keratin-14 (basal epidermal marker) positive iPSC-derived keratinocytes were also able to participate in melanin transfer.

### Generation of 3D skin equivalents from iPSC-derived fibroblasts, keratinocytes and melanocytes

**Fig 5:**
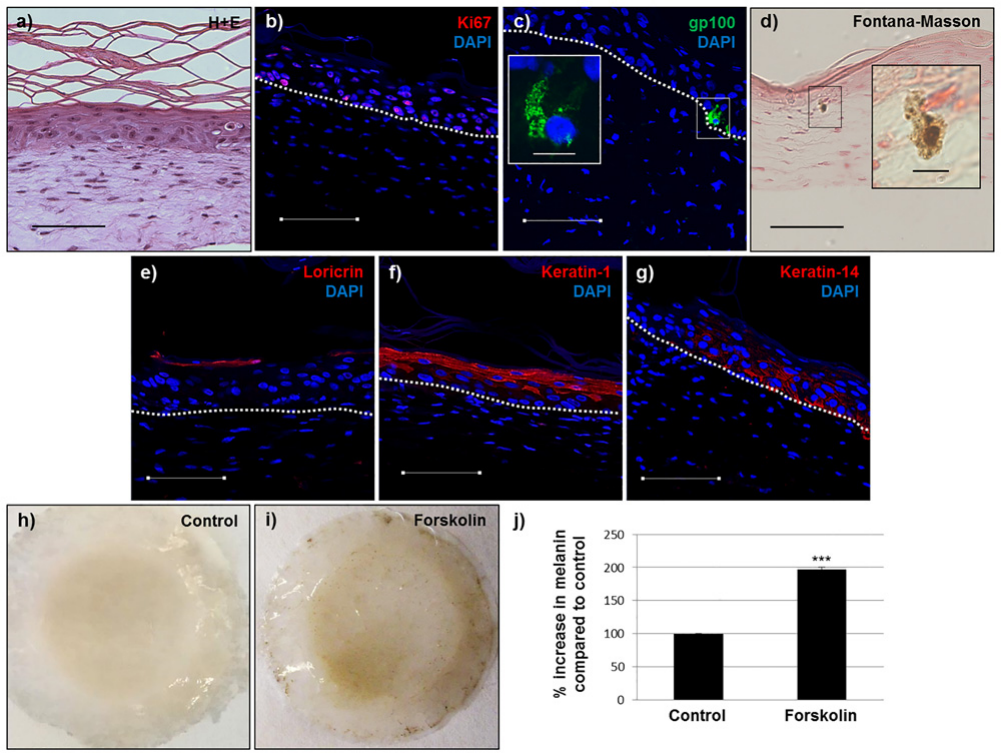
3D skin equivalents produced from iPSC-derived fibroblasts, keratinocytes and melanocytes have normal architecture and are functional. **a)** Hematoxylin and eosin, **b)** Ki67, **c)** gp-100, **d)** Fontana-Masson, **e)** loricrin, **f)** keratin-1, **g)** keratin-14. Blue (DAPI), dashed line (basement membrane). Inset c) (enlarged image showing an iPSC-derived melanocyte in the basal layer of the epidermis). Inset d) (enlarged image showing an iPSC-derived melanocyte producing melanin). **h & i)** Control and forskolin-treated iPSC-derived 3D skin equivalents, respectively, after 14 days at the air-liquid interface. **j)** Quantification of melanin increase in 3D skin equivalents in response to 40μM forskolin. Scale bar = 100μm (except in enlarged images = 10μm). *** = p<0.001.

As reported previously, we have successfully generated 3D skin equivalents from iPSC-derived fibroblasts and keratinocytes [14]. In order to determine whether our iPSC-derived melanocytes could be incorporated into this model, we generated 3D skin equivalents from iPSC-derived fibroblasts, keratinocytes and melanocytes and tissue architecture and cellular localization were determined by hematoxylin and eosin (H+E) staining, Fontana-Masson staining and immunofluorescence. After 2 weeks at the air-liquid interface 3D skin equivalents consisting of iPSC-derived fibroblasts, keratinocytes and melanocytes exhibited normal tissue architecture as determined by H+E staining (Fig 5a) with a stratified and differentiated epidermis as determined by loricrin, keratin-1 and keratin-14 staining (Fig 5e–5g). Additionally, cells in the basal layer of the epidermis remained viable as determined by Ki67 staining (Fig 5b). iPSC-derived melanocytes localized to the basal layer of the epidermis, extended dendrites into the suprabasal layers of the epidermis, exhibited the characteristic punctate staining pattern of melanosomes as determined by gp100 staining and produced melanin as confirmed by Fontana-Masson staining (Fig 5c and 5d). In order to determine whether our iPSC-derived 3D skin equivalents were functional with regards to pigmentation, we added 40μM forskolin (a known pro-pigmentary agent, [26]) to the culture media for 21 days. Exposure to forskolin under these conditions increased levels of melanin in the constructs above those in controls by about 100% (Fig 5h–5j).

## Discussion

Previously, we reported generation of 3D skin equivalents composed exclusively of human iPSC-derived keratinocytes and fibroblasts demonstrating that iPSCs can provide the basis for modeling a human organ derived entirely from two different types of iPSC-derived cells [14]. We report here the generation of the first 3D skin equivalents consisting of human iPSC-derived fibroblasts and keratinocytes as well as melanocytes, and containing functional epidermal-melanin units. To our knowledge, this is the first report of 3D skin equivalents generated exclusively from 3 human iPSC-derived cell types, and the first description of melanin generation and transfer between human iPSC-derived melanocytes and keratinocytes.

In order to generate iPSC-derived fibroblasts and keratinocytes, we used our previously reported differentiation protocols [12,14]. In order to generate iPSC-derived melanocytes, we modified existing differentiation protocols [18,20] as we could not efficiently differentiate our iPSC lines into melanocytes using these protocols in their present form. Moreover, we attempted to utilize an additional melanocyte differentiation protocol [33] and this was also somewhat unsuccessful. This suggests that there may have been intrinsic variability in the capacities of the different iPSC lines. The different methods used for reprogramming of somatic cells into a pluripotent state is one of the most likely reasons for this variability [34] and is an issue which must be addressed fully before the generation of organs solely from multiple iPSC types can be readily replicated in different laboratories that do not have access to the same iPSCs. The protocol we utilized in order to generate iPSC-derived melanocytes was clearly successful, as indicated by the presence of multiple melanocyte specific markers and melanin production in these cells. However, it must be noted that since we initiated differentiation of iPSCs by generating embryoid bodies it is likely that the efficiency of our protocol was low.

To generate a functional epidermal-melanin unit, keratinocytes must internalize melanosomes. Our iPSC-derived keratinocytes internalized melanosome-sized particles in a 6 hour period at a rate that was not significantly different from normal keratinocytes. Whether this similarity in uptake would remain at later time points, remains to be investigated. Also, the rate of uptake we observed in our study was not directly comparable to that observed in a similar study [22] most likely because of the different fluorescent microsphere concentrations used. The uptake of melanosomes by our iPSC-derived keratinocytes could be inhibited by soybean trypsin inhibitor (STI) to an extent similar to that observed in normal keratinocytes. We report that after 24 hours incubation with 1mg/ml STI, melanosome uptake by iPSC-derived keratinocytes was inhibited by about 50%. This compares well with the approximately 60% inhibition observed by others [23] when they incubated normal keratinocytes with 1mg/ml STI for 48 hours. Both our iPSC-derived keratinocytes and normal keratinocytes were derived from Caucasian skin to normalize any effect of skin color on potential melanin uptake. Therefore, whether any differences in melanin uptake or inhibition of uptake would exist under these experimental conditions between normal and iPSC-derived keratinocytes from different skin types also remains to be investigated. It is noteworthy that others [22] found no difference in melanosome-sized particle uptake between normal keratinocytes from different skin types in the time period used in our study.

Notably, K14 positive keratinocytes can transfer melanin. This result shows keratinocytes are closely interacting with melanocytes, and involved in skin pigmentation and UV protection. One of the essential functions of a keratinocyte is to uptake and transfer melanin. This finding illustrates iPSC-derived keratinocytes exhibit this essential function. Interestingly, we noted that some iPSC-derived keratinocytes appeared to internalize greater amounts of melanin when incubated with melanosomes, compared to normal keratinocytes. However, we did not examine this quantitatively. It is possible that our populations of iPSC-derived keratinocytes were heterogeneous and contained cells from multiple stages of keratinocyte differentiation. Therefore, some cells within the iPSC-derived population may have had an increased capacity to internalize melanin than the normal cells which were almost exclusively basal keratinocytes. It is of note that PAR-2 expression, an important regulator of melanosome uptake in keratinocytes, has been shown to be increased in suprabasal compared to basal human epidermis [35]. Nevertheless, we demonstrated melanosome uptake and perinuclear distribution in both K1 and K14 positive iPSC-derived keratinocytes in 2D co-culture with iPSC-derived melanocytes. These findings highlight another obstacle that must be overcome before iPSC-derived organs can be consistently reproduced and the potential benefit of utilizing purification steps at the end of iPSC differentiation protocols.

3D skin equivalents comprised of iPSC-derived fibroblasts, keratinocytes and melanocytes exhibited relatively normal tissue architecture. Importantly, iPSC-derived melanocytes homed to the basal layer of the epidermis and produced melanin in this localization. The ratio of melanocytes to keratinocytes in the basal layer of the epidermis is about 1:10 [36]. However, although iPSC-derived melanocytes were added to the skin equivalent in an amount that has previously been shown to result in physiologically comparable numbers of melanocytes in the basal layer of the epidermis [37] we found them somewhat underrepresented in our models. This finding is unlikely to be due to technical issues during skin reconstruction, as we regularly produce models from freshly isolated somatic cells that do not exhibit this anomaly, but instead may suggest that our populations of iPSC-derived melanocytes were heterogeneous. A proportion of cells produced during our differentiation protocol may not have been of the melanocyte lineage, possibly due to the utilization of embryoid bodies. Additionally, a proportion of cells may not have been sufficiently mature to be capable of homing to this physiological niche. Either way, purification steps at the end of our iPSC differentiation protocol would have been beneficial. We indirectly assessed the viability of our iPSC-derived melanocytes by successfully expanding them in 2D culture and passaging them multiple times in iPSC-derived melanocyte media. However, we did not assess their viability in our 3D skin equivalents. It may be possible that a change in media and a transition from a 2D to a 3D environment impacted negatively on our iPSC-derived melanocyte viability. Consequently, this may also help explain the lower-than-expected iPSC-derived melanocyte numbers found in our skin equivalents. Nevertheless, our iPSC-derived 3D skin equivalents were functional in that the level of melanin could be increased by incubation with a known melanogenic stimulator i.e. forskolin.

In summary, our study demonstrates the generation of functional pigmented human 3D skin equivalents consisting solely of iPSC-derived cell types. This is an important milestone in the generation of complex skin models that more fully recapitulate normal human skin and yet consist of cells from a single individual, a much needed tool required for drug discovery in the era of personalized medicine. Moreover, it highlights the necessity for future optimizations of the global protocol associated with iPSC-derived organs that will be needed for downstream applications.

## Supporting Information

S1 Fig Schematic representation of iPSC–melanocyte differentiation protocol. Media 1 (KO-DMEM supplemented with 20% KO-Serum Replacement, 1% GlutaMax-I, 1% nonessential amino acid, 1% penicillin-streptomycin and 4ng/ml FGF_2_), Media 2 (KO-DMEM supplemented with 20% KO-Serum Replacement, 1% GlutaMax-I, 1% nonessential amino acid, 1% penicillin-streptomycin, 500nM LDN193189 and 10μM SB431542), Media 3 (50% KO-DMEM supplemented with 20% KO-Serum Replacement, 1% GlutaMax-I, 1% nonessential amino acid and 1% penicillin-streptomycin and 50% Neurobasal Media with 2% B-27 Supplement, 2% N-2 supplement, 1% GlutaMax-I, 100nM EDN3, 25ng/ml BMP4 and 50ng/ml SCF), Media 4 (Neurobasal Media with 2% B-27 Supplement, 2% N-2 supplement, 1% GlutaMax-I, 100nM EDN3, 25ng/ml BMP4 and 50 ng/ml SCF), Media 5 (Media 4 + 500μM dbcAMP). 3μM CHIR99021 was added continuously from day 2 of the differentiation protocol onwards.
(TIF)

S1 Table Primers used to determine melanogenic gene expression in iPSC-derived melanocytes. (DOCX)
