# Supplementary figures and images for: Melanin Transfer in Human 3D Skin Equivalents Generated Exclusively from Induced Pluripotent Stem Cells
Source: PLoS One. 2015 Aug 26;10(8):e0136713. doi: 10.1371/journal.pone.0136713 (PMC4550351; doi:10.1371/journal.pone.0136713)

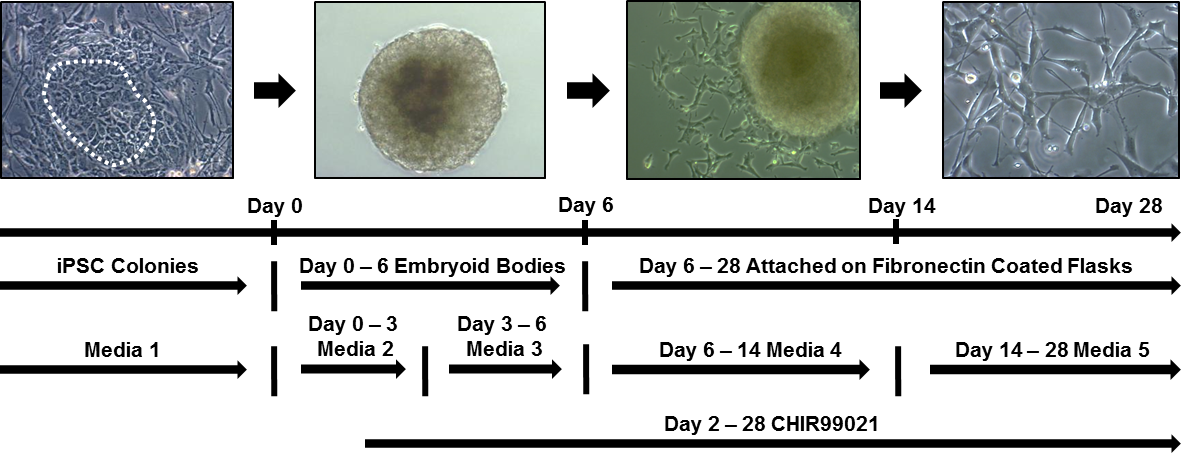

Supplement: S1 Fig — Media 1 (KO-DMEM supplemented with 20% KO-Serum Replacement, 1% GlutaMax-I, 1% nonessential amino acid, 1% penicillin-streptomycin and 4ng/ml FGF2), Media 2 (KO-DMEM supplemented with 20% KO-Serum Replacement, 1% GlutaMax-I, 1% nonessential amino acid, 1% penicillin-streptomycin, 500nM LDN193189 and 10μM SB431542), Media 3 (50% KO-DMEM supplemented with 20% KO-Serum Replacement, 1% GlutaMax-I, 1% nonessential amino acid and 1% penicillin-streptomycin and 50% Neurobasal Media with 2% B-27 Supplement, 2% N-2 supplement, 1% GlutaMax-I, 100nM EDN3, 25ng/ml BMP4 and 50ng/ml SCF), Media 4 (Neurobasal Media with 2% B-27 Supplement, 2% N-2 supplement, 1% GlutaMax-I, 100nM EDN3, 25ng/ml BMP4 and 50 ng/ml SCF), Media 5 (Media 4 + 500μM dbcAMP). 3μM CHIR99021 was added continuously from day 2 of the differentiation protocol onwards. (TIF) [file pone.0136713.s001.tif]
