# Supplementary material for: Melanin Transfer in Human 3D Skin Equivalents Generated Exclusively from Induced Pluripotent Stem Cells
Source: PLoS One. 2015 Aug 26;10(8):e0136713. doi: 10.1371/journal.pone.0136713 (PMC4550351; doi:10.1371/journal.pone.0136713)
Supplement: S1 Table — (DOCX) [file pone.0136713.s002.docx]

| **Primer Name** | **Forward (5’ - 3’)** | **Reverse (5’ - 3’)** | **Product Size (bp)** |
| --- | --- | --- | --- |
| GAPDH | GGGAGCCAAAAGGGTCATCAT | CATGAGTCCTTCCACGATACC | 184 |
| MITF-M | TCTACCGTCTCTCACTGGATTGG | GCTTTACCTGCTGCCGTTGG | 142 |
| DCT | GGTCCCTACATCCTACGAAA | ATGTTCTGCCGAATCACTGG | 173 |
| TYR | GGAGGTCAGCACCCCACAAAT | CATGGTTTCCAGGATTACGCC | 157 |
| MLANA | GGGAGTCTTACTGCTCATCG | CATAAGCAGGTGGAGCATTGG | 203 |
